# Thermal Ablation of Small Renal Tumors – Present Status

**DOI:** 10.1100/tsw.2007.144

**Published:** 2007-02-09

**Authors:** Jon A. J. Lovisolo, Claudio P. Legramandi, Aldo Fonte

**Affiliations:** Department of Urology, Ospedale Galmarini, Piazza Zanaboni, 1, Tradate, Italy

**Keywords:** renal tumors, renal cell carcinoma, ablation, energy ablation, cryotherapy, radiofrequency

## Abstract

Thermal ablation of renal tumors is achieved by the delivery of extreme heat or extreme cold directly to the lesion in order to obtain in situ destruction of the malignant cells without having to remove the entire organ. Cryotherapy and radiofrequency ablation are becoming more and more attractive for the treatment of small lesions in select cases. Other types of energy such as microwave, laser and high intensity ultrasound have also been used to destroy kidney lesions but must still be considered in the experimental stage. Cryotherapy and radiofrequency ablation are minimally invasive and have been shown to be safe and effective in treating tumors up to 3–4 cm in diameter. However, the number of case series is rather limited and follow-up, especially for radiofrequency ablation, is short. Only now are workers beginning to present outcomes after 5 years for cryoablation. Therefore, the long-term oncological efficacy of these ablation techniques remains to be seen. As longer follow-up and greater patient numbers are reported we will get a clearer picture of the true potential of these modalities. Randomized prospective trials would be auspicable. For now, CA and RFA should be limited to few select patients i.e. patients with comorbidities which render them at high risk for a surgical procedure and possibly patients with genetic conditions such as Von Hippel Lindau disease who will probably develop multiple tumors.

## INTRODUCTION

Since 1963 when Robson[1] described the principles of radical nephrectomy, renal oncological surgery has evolved to include new types of procedures and new concepts. The gold standard tenets of renal artery ligation before mobilization of the organ and wide excision including removal of the entire kidney with its perirenal fat as well as removal of the adrenal gland are now generally felt to be unnecessary in some cases. Partial nephrectomy for small tumors has become commonly accepted practice among urologists worldwide. This was first performed in patients with absolute indications for nephron sparing surgery (NSS) such as missing contralateral kidney but slowly, even though the outcomes of partial and radical nephrectomies have never been compared in a randomized trial, NSS has become generally accepted as proper treatment even in patients with bilateral, normally functioning kidneys. Furthermore, the 4 cm tumor diameter cutoff once thought of to be the acceptable limit to elective partial nephrectomy is being extended to include selected patients with tumors up to 7 cm in diameter[2].

Advances in technology and increased experience have promoted the emergence of the laparoscopic approach to renal surgery and many centres currently practice both radical and partial nephrectomies laparoscopically either through a transperitoneal or retroperitoneal approach.

All the above-mentioned modalities are extirpative i.e. their objective is complete tumor removal. However, technological advances now allow the application of energy sources directly to certain types of tumors for *in situ* ablation whose objective is to destroy the lesion without having to remove it. These treatment modalities are all based on the delivery of energy to the renal lesion in such a way that this energy causes temperature changes within the lesion which are sufficient to create tissue necrosis. The latter may be produced either by extreme heat or extreme cold, but the end result is the same i.e. cell death. The challenge of any energy based ablative technique is to create homogeneous and complete tissue necrosis which encompasses the entire renal lesion but spares as much healthy surrounding tissue as possible. Furthermore, the ablative energy must be able to be delivered in a predictable and reproducible fashion without creating damage to surrounding organs or vital renal structures such as the collecting system and vessels. Modalities such as cryotherapy, radiofrequency ablation, microwave and laser ablation are most commonly applied via probes which are inserted into the lesion through laparoscopic or percutaneous approaches. High intensity focused ultrasound, on the other hand, may be delivered extracorporeally in non-invasive fashion.

## CRYOABLATION

Cryoablation has received more study than all the other ablative techniques. The mechanism of cell destruction through freezing is not completely known. Acute and delayed types of tissue damage have been described. The former is due to ice crystal formation within the extracellular space, which creates a hyperosmotic environment thereby causing cell dehydration and shrinkage, enzyme denaturation, and dysfunction of the cytoskeleton and membrane. Cryotherapy therefore initially produces coagulation necrosis and ultimately fibrosis and scarring[3].

Cryoprobes utilize compressed gas, generally argon or nitrogen, to produce tissue temperatures as low as –190°C. It has been shown that complete ablation of renal tissue in pigs was uniformly produced at temperatures of -19.4C or lower however, it may be desirable to achieve tissue temperatures of at least –40°C as is done in prostate cryotherapy and most clinicians strive to achieve this temperature. From the center of the ice ball to its edge temperature rises rapidly towards 0°C so it is important to maintain margins of at least 3.5 and up to 10 mm beyond the tumor outer edge in order to guarantee sufficiently low temperatures throughout the entire tumor. Real time ultrasound is generally used to monitor the extension of the ice ball or alternatively, tissue temperature can be directly measured through thermocouples placed at the tumor margin. Double freeze thaw cycles are generally employed to ensure more efficacious tissue destruction within the ice ball while not increasing the size of the cryolesion. Thawing may be passive or active and it is still not clear which of the 2 modalities produces better results[4,5].

Cryoprobes range in diameter from 1.7 to 5 mm and are inserted into the center of the tumor through open surgical, laparoscopic or percutaneous approaches. Open surgery, however, is rarely used since the trauma of the procedure would defeat one of the main reasons for performing cryosurgery, i.e. minimal invasiveness.

Laparoscopic renal cryoablation is now offered in many urological centers. This approach is advantageous in that it allows precise probe placement, direct real-time ultrasound monitoring of the ice ball formation and dissection and displacement of adjacent structures from the ablation site. In this way injury to organs such as the ureter, bowel and blood vessels can be avoided. A retroperitoneal or transperitoneal approach can be employed according to the location of the tumor within the kidney to be treated.

Percutaneous cryoabation is also available for the treatment of small renal masses. This is becoming more and more widespread thanks to technological developments which have produced smaller diameter cryoprobes and better and more reliable lethal isotherms as well as better imaging techniques. However, this approach presents several limitations in comparison to laparoscopic cryoablation. Access to anterior tumors is difficult making percutaneous cryoablation feasible essentially only for posterior and lateral lesions. Percutaneous renal cryoablation is difficult to monitor during the procedure since direct visualization of the ice ball as in laparoscopy is impossible and ultrasonography is often ineffective due to the interference of intervening anatomical structures. Thus, the percutaneous approach to cryoablation often must rely on CT or open gantry MRI for intraoperative image guidance and ice ball formation monitoring[6] rendering it difficult to perform in many centers.

## LAPAROSCOPIC RENAL CRYOABLATION

Gill and coworkers[7] reported on their initial series of 11 renal tumors in 10 patients treated by cryoablation through a retroperitoneoscopic approach. The tumors were all exophytic and ranged in size from 1.5 to 3 cm as measured by computed tomography. Tumors were located at the upper (3), middle (5), or lower (3) pole of the kidney. A 3-port retroperitoneal laparoscopic approach was used. Puncture cryoablation was performed with a 4.8-mm cryoprobe. Real-time, endoscopic, steerable, color Doppler ultrasound was used to monitor the evolving cryolesion. The ice ball was intentionally created up to 1 cm beyond the tumor edge with the aim of achieving negative margins. Mean surgical time was 2.4 hours, cryoablation (double freeze-thaw) time 12.9 minutes, cryoprobe tip temperature -186 degrees C, and blood loss 75 mL. Systemic temperature remained unaltered. Hospital stay was less than 23 hours in 9 of 10 patients. Follow-up magnetic resonance imaging at 1 day and 1, 2, and 3 months identified the punched-out, nonenhancing, spontaneously resorbing, renal cryolesion. Postablation biopsies performed in 3 patients at 3 to 6 months after surgery were all negative for tumor.

The same authors recently reported on 56 patients who underwent laparoscopic renal cryoablation and who had completed a followup of at least 3 years each[8]. Serial magnetic resonance imaging (MRI) was obtained at 1 day, months 1, 3, 6, 12, 18 and 24, and yearly thereafter for 5 years. Computerized tomography guided needle biopsy of the cryolesion was performed 6 months postoperatively and repeated if MRI findings were abnormal. The mean tumor size was 2.3 cm and the mean intraoperative size of the created cryolesion was 3.6 cm. There was seen to be a 75% mean reduction in lesion size 3 years postoperatively and at 3 that time 17 cryolesions (38%) had completely disappeared on MRI. Postoperative needle biopsy identified locally persistent/recurrent renal tumor in 2 patients. In the 51 patients undergoing cryotherapy for a unilateral, sporadic renal tumor 3-year cancer specific survival was 98%. There was no open conversion, kidney loss, urinary fistula, dialysis requirement, or perirenal or port site recurrence in any patient.

Desai et al[9] retrospectively compared the short term results of laparoscopic partial nephrectomy to laparoscopic cryoablation and found that the partial nephrectomy patients were generally younger and healthier with larger tumors, had lower baseline creatinine levels, and had fewer solitary kidneys. Laparoscopic partial nephrectomy was associated with higher average blood loss and more late complications while intraoperative and early complication rates were similar as were operative time, hospital stay, convalescence and postoperative creatinine. They conclude that the technical simplicity and safety profile of laparoscopic cryoablation render it a very attractive procedure, but caution must be exercised since long term oncological results are not yet available.

Cestari et al[10] published their results of laparoscopic renal cryoablation in 37 patients with image documented solid lesions. The mean tumor diameter was 25.7 mm (range 10 to 60). Mean operative time was 194 minutes and mean blood loss was 165 ml. Histology showed renal cell carcinoma in 29 cases and oncocytoma in 6, while 2 were reported as indefinite. Mean diameter of the cryolesion on postoperative day 1 on MRI was 48.2 mm and it progressively decreased during followup. Surgical complications included: transient hyperthermia in 3 cases, hematoma in 2 patients, hematuria in 1 patient and UPJ

obstruction which required pyeloplasty 8 months after cryoablation in 1 patient. Twenty five patients underwent CT guided biopsy after 6 months of follow up and all of these were negative for neoplasia.

Moon et al.[11] employed a double freeze – active thaw technique to perform laparoscopic renal cryoablation on 16 patients with tumors of a mean diameter of 2.6 cm. After a mean follow- up period of 9.6 months all the lesions were smaller or stable and none were enhancing.

Davol et al[12] have published their long term results with laparoscopic renal cryoablation . They retrospectively identified 48 patients, with a median follow-up of 64 months (range, 36-110 months) and an overall survival rate of 89.5%. The median size of the treated lesions was 2.6 cm (range, 1.1-4.6 cm). A total of 12.5% of patients were diagnosed with persistent disease during the follow-up period. The cancer-specific survival rate was 100%, and the cancer-free survival rate after a single cryoablation procedure was 87.5%. This improved to 97.5% after a repeat procedure. No major complications were observed.

## PERCUTANEOUS RENAL CRYOABLATION

Percutaneous renal cryoablation was first described in 1995 by Uchida and coworkers[13]. These authors used a specially designed 6.8mm cryo probe under ultrasound guidance to treat 2 patients with metastatic renal cell carcinoma. They performed renal artery embolization prior to cryoablation. Postoperative shrinkage of the entire kidney was observed in both cases and the patients' Karnofsky performance status showed a marked improvement 3 months after the operation, but their general condition gradually deteriorated. The patients died 10 months 5 months after the procedure. These authors postulated that cryo-immunological activity had contributed to the temporary improvement in their condition.

Shingleton and Sewell[14] performed MRI guided percutaneous cryoablation on 22 tumors in 10 patients. Their protocol called for treating only tumors of 4 cm or less but two patients with large tumors and who were otherwise inoperable were treated on a compassionate basis. Under MRI guidance a 2 or 3 mm cryoprobe was advanced into the renal mass and the mass was subjected to 3 freeze-thaw cycles at -80 to 70C. Patients were hospitalized overnight for observation. Tumor sizes ranged from 1.8 to 7 cm with a mean diameter of 3cm. After a mean followup of 9 months, one patient required retreatment for evidence of persistent tumor.

Harada et al[15] performed percutaneous cryoablation on radiographically documented masses of 4 cm or less in diameter (mean 2.5 cm). Tumor location was at the upper pole in two cases and midkidney in two cases. Cryoablation was performed by two freeze, active thaw cycles, and the cryoprobes were withdrawn or advanced between the cycles when necessary. Freezing time was less than 20 min/cycle mainly depending on the size of the iceball. Thawing time was less than 10 min/cycle. Two or 3 mm diameter cryoprobes were used and up to 4 probes were placed during the procedure. One case of perinephric hemorrhage not requiring surgery was observed. Short term follow up of these patients revealed persistent tumor in 1 case which required retreatment 6 weeks after the initial procedure.

Bassignani et al[16] treated three patients with 4 enhancing renal masses in 4 kidneys. Renal cryoablation was performed using general anesthesia with 17 gauge cryoneedles percutaneously placed into the renal tumor under real-time sonographic guidance. No perioperative complications were noted. All patients were discharged home within 24 hours of the procedure and postoperative pain was controlled with oral nonnarcotic medications. Followup cross-sectional imaging performed 6 to 7 weeks following cryoablation indicated that the lesions shrank an average 63% +/- 15% from initial pretreatment volume. Importantly, none of the lesions showed contrast enhancement by computerized tomography or magnetic resonance imaging criteria.

Gupta et al[17] performed CT guided percutaneous renal cryoablation on 27 tumors using conscious sedation in 20 patients. Only 1 hemorrhagic complication occurred requiring blood transfusion in a patient with a large, centrally located tumor. Sixteen tumors in 12 patients with a mean followup period of 5,9 months received imaging followup. Mean baseline tumor size in this group was 2.5 cm with 11 small (3 cm or less) and 5 large (more than 3 cm) tumors. Of the tumors 5 were centrally located and 11 were noncentrally located. Preliminary data suggest that of the 16 cryoablated tumors 15 showed no signs of enhancement on followup.

## RADIOFREQUENCY ABLATION

Zlotta et al[18] first described radiofrequency ablation of renal tumors in 1997. These authors first performed an *ex-vivo* experiment on 4 human kidneys just after their removal. Bipolar RF energy applied to the kidneys in the ex-vivo experiment produced maximum temperatures at the active needles which ranged from 84°C to 130° C. Lesions were on average 2.2 x 3 x 2.5 cm.3 Microscopic examination showed stromal edema with intensive pyknosis and no damage was seen to adjacent untreated tissue. They then treated 4 patients – 3 had RF ablation of tumors under general anesthesia just before undergoing nephrectomy and 1 patient with a 2 cm peripheral lesion was treated percutaneously under local anesthesia 1 week prior to radical surgery. In the *in vivo* procedure, tolerance of radiofrequency ablation as an anesthesia-free procedure was excellent. The size of the observed RF lesions was comparable to the forecast size. No side effects were noted, and no adjacent structures were affected by the RF ablation. These preliminary studies demonstrated the ability to percutaneously deliver radiofrequency energy to produce localized extensive necrosis in kidney parenchyma and tumors safely under local anesthesia and opened the door to the subsequent development and clinical employment of the procedure.

Radiofrequency energy is delivered to the tumor by means of an electrode in the form of a thin (14 – 18 gauge) needle which is electrically insulated along its shaft except for the distal 1 to 3 centimeters. The needle is placed percutaneously into the tumor under US guidance and then connected to a generator which causes the emission of RF energy from the distal part of the needle. Bipolar and monopolar systems are available. The RF probes create tissue temperatures of up to 105°C. Cell death and coagulation necrosis generally occur at 70°C. Conventional monopolar RF ablation is limited by the fact that tissue vaporization and charring occur at the probe tip. This raises impedence therefore limiting heat dispersion to approximately 1.6cm. To overcome this limitation more efficient probes have been designed[19]. These include internally cooled electrodes, multitined expandable electrodes and perfusion electrodes. An internally cooled electrode uses cooled saline circulation within the probe itself to keep the distal end at a constant temperature. This avoids tissue charring and therefore permits better heat diffusion to the surrounding tissues. Multitined expandable electrodes, also known as umbrella electrodes, Christmas tree electrodes, multiple hooked electrodes or arrays, contain a series of retractable electrode tines which are deployed from the tip of a single centrally positioned larger needle cannula. The tines act as multiple miniprobes allowing the creation of larger and more uniform lesions. Perfusion electrodes have small apertures at the active tip that allow fluids (normal or hypertonic saline) to be infused or injected into the tissue before, during, or after the ablation procedure. In so called wet RFA hypertonic saline is perfused into the tissue surrounding the probe tip thereby spreading the current density and heat away from the tip.

Real time ultrasound is generally employed during RFA in order to monitor the developing lesion. The lesion appears hyperechoic upon ultrasound, however the margins are irregular and accurate visualization of the expanding lesion difficult[20].

Recently, Johnson et al[21] have shown that contrast-enhanced ultrasonography appeared to be an accurate modality for immediate monitoring of RFA defects. Further work by Slabaugh et al[22] showed that RFA lesions could not be imaged accurately in real time with standard gray-scale or Doppler sonography, but microbubble contrast enhanced ultrasound was able to monitor parenchymal blood flow and, thus, the lesions (absence of blood flow) in real time. The hypoechoic areas without bubble enhancement imaged during contrast sonography corresponded with regions of cell death as demonstrated on pathologic analysis.

Two different systems are used to monitor RFA lesion growth and extension: impedence and temperature monitoring. Animal studies have suggested that the latter is more accurate due to the fact that impedence based RF caused incomplete necrosis with skip areas[20].

## RADIOFREQUENCY ABLATION – CLINICAL RESULTS

Pavlovich et al[23] performed RFA on 24 percutaneously accessible renal tumors in 21 patients. Tumors were 3 cm. or less in diameter and solid on computerized tomography. After 2 months of follow-up mean tumor diameter decreased from 2.4 to 2.0 cm, and a majority of tumors (19 of 24, 79%) ceased to be enhanced on contrast CT. Mean serum creatinine remained stable during this interval. No major and 4 minor complications were encountered, including 2 episodes each of transient psoas pain and flank skin numbness.

Ogan[24] and coworkers reported on percutaneously administered, CT guided RFA in 12 patients with 13 small (less than 4 cm) posterior or lateral contrast-enhancing renal tumors. The mean tumor size in this series was 2.4 cm. The average procedure time was 95 minutes and length of stay 1day. There were no major complications. After a mean follow-up of 5 months, 12 (93%) of 13 tumors had been successfully ablated.

Jacomides et al[25] performed laparoscopic radio frequency ablation in 13 patients with a total of 17 (1 patient with 5 lesions) small enhancing renal masses. In 5 patients the tumor was surgically removed after treatment, whereas in 7 it was left in situ. Mean tumor size was 2 cm. (range 0.9 to 3.6). None of the 8 patients with renal cell carcinoma who had at least 6 weeks of followup (mean 9.8 months) had any evidence of persistent tumor enhancement on surveillance computerized tomography or any other evidence of disease progression. There was 1 focal positive margin in a patient who underwent radio frequency ablation and excision of renal cell carcinoma but the patient remained disease-free 1 year after treatment.

Hwang[26] treated 17 patients with a total of 24 renal tumors ranging from 1.2 to 2.85 cm with RFA under laparoscopic (9) or percutaneous (8) guidance. The laparoscopic approach was used if the tumors were contiguous to bowel, ureter or large vessels. Postoperative followup consisted of contrast CT at regular intervals. They found that at a median followup of 1 year, median tumor or thermal lesion diameter decreased from 2.26 to 1.62 cm, and only 1 lesion (4%), which was located centrally near the hilum, exhibited contrast enhancement on CT at 12 months. In 1 patient whose ureter was adherent to the tumor a ureteropelvic junction obstruction developed after laparoscopic RFA, requiring open repair.

Zagoria et al[27] performed 27 CT guided, percutaneous renal RF ablations in 22 patients. These authors employed an impedence based generator with cooled probes. The type of probe varied according to tumor size: tumors <1 cm were treated with a 2 cm single tip probe; tumors from 1-2 cm were treated with a 3 cm tip and those above 2 cm were treated with a 3 electrode cluster array. Mean tumor size was 3.5cm. They found that complete tumor ablation was achieved after a single treatment session in 83% of patients, and in 8% of patients after subsequent ablation sessions. After a mean follow-up period of 7 months, 20 of 22 patients (91%) were found to be free of disease as determined by contrast CT or MRI. These authors found that size was the major determinant for achieving tumor eradication with a single session of ablation, with all 11 tumors 3 cm or smaller being completely ablated after one session. Tumor location, histology, and the presence of renal disease did not correlate with treatment success.

Conversely, Permpongkosol et al[28] found that treatment success was more dependent upon tumor location than upon size. They reported on a series of 21 patients with 23 biopsy proven renal cell carcinomas who underwent CT guided cryoablation. In agreement with Zagoria et al[27] they found that that tumors greater than 3 cm in diameter required multiple probes and had increased likelihood of incomplete treatment or recurrence. However, since larger tumors can be treated with more probes, they felt that this was not the primary determinant of success. In their experience, of the 6 patients who had tumors located centrally, 3 patients (50%) required a second session of cryoablation and 2 of them were never determined free of enhancement. On the other hand, 16 of 17 tumors classified as noncentral

(94.1%) had no imaging evidence of residual viable tumor after a single ablation session. These authors identified 2 likely factors that make central tumors more difficult for ablation. The first is the presence of large blood vessels near the hilum which limit temperature excursions in the surrounding tissues by the creation of a heat sink through the continuous delivery of body temperature blood. The second major factor was felt to be the difficulty in achieving tumor-free margins without harming the nearby central structures.

Varkarakis et al[29] have published their results with RFA for renal tumors in a series of 49 patients with 60 tumors. Among these, there were 46 evaluable patients (56 tumors) with more than 2 years of mean follow-up. Six tumors were incompletely treated with the first RFA and successfully treated with a second session. Recurrences after successful initial treatment were seen in 3 of 46 patients. These recurrences all occurred in patients with a central tumor of 3.0 cm or greater. Overall local control was achieved in 95% of tumors.

Lewin and coworkers[30] published the initial results they obtained with direct MRI guided RFA in a series of 10 patients with small exophytic kidney tumors (mean diameter 2.3 cm). Complete ablation, as defined by MR imaging, was achieved in all cases by the end of the procedure. No intra- or postprocedural complications aside from two small self-limited perirenal hematomas were observed. No delayed complications or tumor recurrence occurred during a mean follow-up period of 25 months.

Hegarty et al[31] treated 82 tumors by percutaneous CT guided RFA in 72 patients. Mean tumor size was 2.51 cm. MRI performed on the first postoperative day showed findings compatible with persistent malignancy in 4% of patients (3 cases). Two of these were retreated successfully. The other patient was followed and was seen to be stable. During followup an additional 5 patients required retreatment for recurrent disease for an overall retreatment rate of 11%.

## COMPLICATIONS OF CRYOTHERAPY AND RADIO FREQUENCY ABLATION

RFA and cryoablation for renal tumors have been seen to be safe procedures with a low percentage of intraoperative and postoperative complications. The procedures are well tolerated and in many centers they are performed with only conscious sedation. Interestingly, Allaf et al.[32] retrospectively compared the analgesic and sedative requirements in patients undergoing percutaneous renal cryoablation vs. percutaneous RFA and found that cryoablation was associated with a significantly lower use of analgesia. No patients in the cryoablation group required additional or alternate anesthetics while 3 patients in the RF group required additional drugs and one patient required early termination of the procedure due to pain.

Approximately 40% of the patients who undergo percutaneous RFA experience low-grade fever while as many as 80% report flu-like symptoms. The combination of these two, the post-radiofrequency ablation syndrome, is seen in approximately 1/3 of treated patients. Complete post-RF ablation syndrome is generally self-limiting and resolves within 10 days but , persistent or late-onset fever may be a sign of concurrent infection elsewhere[33].

A retrospective multi-institution review[34] of complications following percutaneous and laparoscopic RFA and cryoablation found an 11% overall complication rate among which there was a 2% major complication and 9% minor complication rates. There was 1 death which occurred 3 days after percutaneous RFA due to aspiration pneumonia. Overall 87% of the complications were directly attributable to the ablation procedure. The overall complication rate in the cryoablation group was 14% and 8% in the radiofrequency ablation group. The complication rates decreased over time in this series indicating the presence of a learning curve. The most common complication was pain and/or paresthesia at the probe puncture site. This occurred in 5% of cases. Since the entire length of the CA probe is cooled, care must be taken to avoid thermal injury to sensory nerves at the body surface. Only the tip of the RFA probe is active, therefore the heated portion is positioned completely within the kidney but the probe may transmit heat from the treatment site to surrounding tissues. In addition, the temperature based RFA procedure includes track ablation with the intent of decreasing bleeding and needle track seeding, but may also damage cutaneous sensory nerves and lead to probe site pain. Thus, the track ablation mode should only be activated long enough to remove the probe from the kidney and the surrounding Gerota's fascia. In this series, the complication rate in the laparoscopic subgroup was 9% overall with 3% of the complications attributable to laparoscopic technique. One patient who underwent laparoscopic RFA had subclinical urine leakage through the ablated needle tract, which was seen only on retrograde pyelogram. This leakage was self-limited and resolved after a period of observation. Thus, ultrasonographic monitoring has been recommended, whenever possible, to avoid direct calyceal puncture during CA or RFA. Another patient, had UPJ obstruction after laparoscopic RFA for a 2.3 cm tumor near the hilum and renal pelvis. At 6 months followup CT revealed persistent hydronephrosis and delayed contrast excretion and then at 11 months postoperatively nephrectomy was performed.

Small hematomas that remain asymptomatic and do not require transfusion or intervention are common after renal energy ablation and probably should not even be considered to be complications[34]. The rate of minor plus major hemorrhagic complications requiring transfusion or intervention found by Johnson et al (34) in their series was less than 1% (2/271). Measures to reduce the risk of bleeding include RF track ablation as described above and gentle withdrawal of the cryo probe only after complete thawing of the ice ball and application of argon- beam coagulation or bioadhesive hemostatic agents.

A major concern of probe-based ablative techniques is the danger of producing injury to the collecting system during the treatment of lesions that abut the renal pelvis or calices. Brashears et al[35] studied the effects of CA and RFA after deliberate puncture of the collecting system in pigs. They found that after significant intentional injury to the collecting system CA did not produce any urinary fistulae (0/15) while RFA was associated with a high likelihood of urinary extravasation.. These findings are corroborated by the work of Sung et al[36] who intentionally included the pelvicaliceal system within the boundaries of the ice ball, but without direct puncture, during CA in pigs. They compared the effects of CA on the collecting system with and without warm continuous retrograde saline perfusion and found no incidence of urinary extravasation in either group of kidneys. Subsequently, they observed regrowth of normal urothelium with some scarring of the lamina propria or underlying smooth muscle at 1-month, and complete healing of the cryo-injured collecting system with a fibrous scar after 3 months.

Other complications described in the literature include skin metastasis at the needle puncture site following RFA[37], pancreatic injury requiring surgical exploration[38], ileus, and respiratory difficulty[39].

## IMAGING FOLLOW-UP AFTER RENAL TUMOR ABLATION

### Image Type and Characteristics

Contrast medium-enhanced CT or MR imaging are used during follow-up to detect the presence of viable residual or recurrent cancer. This is most frequently found at the margin of the ablation zone. While the CT characteristics of tumor presence in a previously ablated area remain to be clearly defined, Farrell et al[40] arbitrarily defined complete ablation after RF treatment as 10 HU density or less within the thermal lesion. On MRI, low signal of the ablation zone on T2-weighted images and lack of enhancement after gadolinium administration are the general characteristics of completely ablated RCC. Merkle et al[41] prospectively evaluated 18 patients with gadolinium-enhanced MR imaging after RF ablation and found that tumors decreased gradually in size but retained a thin rim of T2-hyperintense tissue, which enhanced with gadolinium. Recurrent tumor was found in 2/18 cases and was best demonstrated on unenhanced T2-weighted and gadolinium-enhanced T1-weighted MR images. Gill et al[42] monitored a cohort of 56 patients with MR imaging after laparoscopic cryoablation and observed a gradual involution in the size of the ablation zone. By 3 years, there was a 75% reduction in cryolesion size, and 38% of cryolesions were undetectable on imaging. Two patients had enhancing areas of nodularity on MR imaging at 6 months, which were biopsy-proven recurrence. Hegarty et al[31] found that while cryoablation lesions shrunk significantly over time, no significant reduction was noted in the size of the radiolesion on the 2 year postoperative MRI scan. Regardless of the type of ablation, lesion growth from one scan to the next or the development of enhancement within the lesion require rebiopsy, and then possibly re-ablation or surgical exploration.

### Frequency of Imaging

The optimal frequency of imaging after thermal ablation is related to the natural history of small RCCs, which is approximately 6 mm growth per year. Most clinicians engaged in thermal ablation of renal tumors perform imaging at 6-month intervals after an early scan (performed as early as 1 week to as late as 1 month), which is necessary to demonstrate the absence of residual enhancing tumor and therefore to exclude the necessity of a repeat treatment. Some perform imaging more frequently within the first year after ablation (every 3–4 months), with the rationale that the first year is when recurrent disease is most likely[43].

## LASER THERMAL ABLATION

It has been shown that high-energy laser radiation produces cogulation necrosis, which is capable of destroying renal tissue. Lofti et al[44] performed an animal experiment in which an interstitial Nd:YAG contact probe produced pronounced tissue vaporization surrounded by a zone of coagulation necrosis approximately 1.5 cm across. These authors concuded that this modality could be used for both controlled coagulation necrosis and vaporization of renal parenchymal tissue.

Dick et al[45] reported on the feasibility of laser thermal ablation of renal tumors in 2002. They treated 9 inoperable patients by laser tumor ablation under MRI guidance and used real time color thermal mapping to monitor tumor ablation. They found that after a mean follow-up of 16.9 months, the tumor size did not change but the mean percentage of viable tumor decreased significantly form 74 to 30%. There were 2 minor and 1 major complications. However, the authors felt the procedure to be safe and feasible.

Very few other clinical results are available in the literature and this type of procedure should be considered purely experimental at the present time.

## HIGH INTENSITY FOCUSED ULTRASOUND

High intensity focused ultrasound (HIFU) is a type of thermotherapy that destroys tissue by coagulative necrosis. Various manufacturers market machines that are capable of delivering this focused energy to tissues transcutaneously. Therefore, it is theoretically possible to treat lesions, including kidney tumors, in an extracorporeal fashion. During HIFU lesion evolution can be followed on ultrasound as a hyperechoic region that starts at the focus of the probe and then builds toward the transducer as the treated tissue reflects the high intensity focused ultrasound waves. This change in acoustical impedance of treated tissue to reflect high intensity focused ultrasound energy back towards the transducer results in the characteristic wedge-shaped high intensity focused ultrasound lesion.

Chapelon et al[46] initially studied in vivo HIFU tissue destruction on 124 rat and 16 canine kidneys with a 1- and 2.25-MHz transducer. In the canine experiments, a kidney lesion was achieved extracorporally in 10 animals. The lesions were found to be characterized by coagulation necrosis. These lesions were felt to be created by a combination of cavitation and thermal effects and depended on the duration and frequency of the ultrasound bursts

Watkin et al[47] performed a feasibility study on HIFU ablation in the porcine kidney. Eighteen kidneys were treated *in vivo* at a depth of 40 mm from the skin surface, with acute damage detected in 13. Damage was macroscopically and histologically discrete and confined to the target area within the kidney. Skin induration was observed after treatment in nine cases, and there was one skin burn. There was wide variation in the extent of renal damage which was believed to have resulted from the narrow acoustical window in the flank, respiratory movement of the nonparalyzed animal and energy attenuation during transmission through the skin and abdominal wall.

The first clinical application of HIFU in man was reported by Valencien in 1993[48]. This group treated 4 patients with T2 to T3 renal tumors with HIFU prior to radical nephrectomy. Histological examination of the treated kidneys revealed coagulation necrosis in the targeted tumor area. A skin burn occurred in 1 patient.

Kohrmann et al[49] performed extracorporeal high intensity focused ultrasound to treat 3 renal tumors in a patient with a solitary kidney.. Three sesions with the patient were required to treat the tumors. Two of the lesions were found to be effectively treated at the 6-month radiological followup, but 1 upper pole lesion was inadequately treated due to ultrasound energy absorption by the interposed ribs.

Marberger et al[50] reported the experience with HIFU for the ablation of renal tumors in 2 institutions. A total of 20 patients were treated. Most of the patients were treated with HIFU pior to nephrectomy, but 2 were treated with curative intent. Some shrinkage but incomplete ablation was seen in one of these patients. Histological examination of the HIFU lesions in the remaining patients whose kidneys were subsequently removed gave findings which varied from no consistent areas of histological damage to acute tissue necrosis which affected no more than 35% of the targeted area. Complications were minor.

Paterson et al[51] recently reported their experimental results on the use of HIFU applied laparoscopically under real time ultrasound visualization. This approach would eliminate the problem of skin burns and could possibly allow the obtaining of more consistent and complete tissue necrosis. They were able to create homogenous and complete tissue necrosis throughout the whole volume of the lesion with sharp demarcation from adjacent normal tissue lesions in 12 of 13 treated kidneys. Three of the 13 animals were excluded from the study due to operative complications which included intraabdominal abscess, sepsis and pneumonia.

HIFU , at the present time, must be considered an investigational means of treatment for renal cell carcinoma. The clinical series reported in the literature are all small with limited follow-up. Furthermore, the immediate results have been disappointing with no study being able to obtain complete and consistent ablation. HIFU cannot be recommended for clinical use at the present time but technological refinements of this modality might render it useful for a subset of patients.

## CONCLUSIONS

Needle based thermal ablation of renal tumors via cryotherapy or radiofrequency is an attractive treatment modality for the small lesion. These 2 types of treatment have been shown to be safe and effective in ablating tumors up to 3 –4 cm in diameter. However, the number of case series is rather limited and follow-up, especially for radiofrequency ablation, is short. Only now are workers beginning to present outcomes after 5 years for cryoablation. Therefore, the long term oncological efficacy of these ablation techniques remains to be seen. As longer follow-up and greater patient numbers are reported we will get a clearer picture of the true potential of these modalities. Randomized prospective trials would be auspicable. For now, CA and RFA should be limited to few select patients – i.e. patients with comorbidities which render them at high risk for a surgical procedure and possibly patients with genetic conditions such as Von Hippel Lindau disease who will probably develop multiple tumors.

Other thermal ablative modalities such as HIFU, laser ablation and microwave ablation have not been sufficiently tested in the clinical setting and therefore should not be considered a viable clinical alternative.
